# Vasomotion loss precedes impaired cerebrovascular reactivity and microbleeds in cerebral amyloid angiopathy

**DOI:** 10.1093/braincomms/fcaf186

**Published:** 2025-05-14

**Authors:** Mariel G Kozberg, Leon P Munting, Lee H Hanlin, Corinne A Auger, Maarten L van den Berg, Baudouin Denis de Senneville, Lydiane Hirschler, Jan M Warnking, Emmanuel L Barbier, Christian T Farrar, Steven M Greenberg, Brian J Bacskai, Susanne J van Veluw

**Affiliations:** Department of Neurology, Massachusetts General Hospital, Boston, MA 02114, USA; Department of Neurology, Massachusetts General Hospital, Boston, MA 02114, USA; Department of Radiology, Leiden University Medical Center, 2333 ZA Leiden, The Netherlands; Department of Neurology, Massachusetts General Hospital, Boston, MA 02114, USA; Department of Neurology, Massachusetts General Hospital, Boston, MA 02114, USA; Department of Neurology, Massachusetts General Hospital, Boston, MA 02114, USA; University of Bordeaux, CNRS, INRIA, Bordeaux INP, IMB, UMR 5251, F-33405 Talence, France; Department of Radiology, Leiden University Medical Center, 2333 ZA Leiden, The Netherlands; Univ. Grenoble Alpes, Inserm, Grenoble Institut Neurosciences, U1216, Grenoble 38000, France; Univ. Grenoble Alpes, Inserm, Grenoble Institut Neurosciences, U1216, Grenoble 38000, France; Department of Radiology, Athinoula A. Martinos Center for Biomedical Imaging, Massachusetts General Hospital, Boston, MA 02114, USA; Department of Neurology, Massachusetts General Hospital, Boston, MA 02114, USA; Department of Neurology, Massachusetts General Hospital, Boston, MA 02114, USA; Department of Neurology, Massachusetts General Hospital, Boston, MA 02114, USA

**Keywords:** cerebral amyloid angiopathy, vasomotion, vascular smooth muscle cells, microbleeds, cerebrovascular reactivity

## Abstract

Cerebral amyloid angiopathy (CAA) is a cerebral small vessel disease in which amyloid-β accumulates in vessel walls. CAA is a leading cause of symptomatic lobar intracerebral haemorrhage and an important contributor to age-related cognitive decline. Recent work has suggested that vascular dysfunction may precede symptomatic stages of CAA, and that spontaneous slow oscillations in arteriolar diameter (termed vasomotion), important for amyloid-β clearance, may be impaired in CAA. To systematically study the progression of vascular dysfunction in CAA, we used the APP23 mouse model of amyloidosis, which is known to develop spontaneous cerebral microbleeds mimicking human CAA. Using *in vivo* 2-photon microscopy, we longitudinally imaged unanesthetized APP23 transgenic mice and wildtype (WT) littermates from 7 to 14 months of age, tracking amyloid-β accumulation and vasomotion in individual pial arterioles over time. MRI was used in separate groups of 12-, 18- and 24-month-old APP23 transgenic mice and WT littermates to detect microbleeds and to assess cerebral blood flow (CBF) and cerebrovascular reactivity (CVR) with pseudo-continuous arterial spin labelling. We observed a significant decline in vasomotion with age in APP23 mice, while vasomotion remained unchanged in WT mice with age. This decline corresponded in timing to initial vascular amyloid-β deposition (∼8–10 months of age), although it was more strongly correlated with age than with vascular amyloid-β burden in individual arterioles. Declines in vasomotion preceded the development of MRI-visible microbleeds and the loss of smooth muscle actin in arterioles, both of which were observed in the majority of APP23 mice by 18 months of age. Additionally, CBF and evoked CVR were intact in APP23 mice at 12 months of age, but significantly lower in APP23 mice by 24 months of age. Our findings suggest that a decline in spontaneous vasomotion is an early, potentially pre-symptomatic, manifestation of CAA and vascular dysfunction, and a possible future treatment target.

## Teaser

In this multi-modal imaging study, Kozberg *et al*. report that the loss of spontaneous oscillations in arteriolar diameter (vasomotion) occurs early in CAA disease progression in the APP23 mouse model. The loss of vasomotion coincided with initial vascular amyloid-β deposition and preceded the occurrence of microbleeds.

## Graphical Abstract

**Graphical Abstract:**
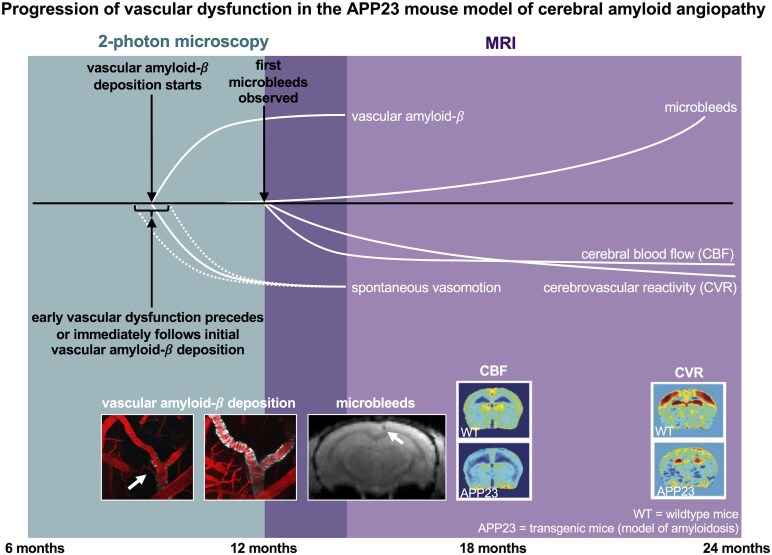

## Introduction

Cerebral amyloid angiopathy (CAA) is a leading cause of intracerebral haemorrhage, a highly lethal type of stroke with a median one-month fatality rate of 40%.^1^ CAA also leads to smaller MRI-visible haemorrhagic lesions in the brain, including cerebral microbleeds and cortical superficial siderosis.^2^ Pathologically, CAA is defined by an accumulation of amyloid-β in the walls of the brain's vasculature, predominantly in cortical and leptomeningeal arterioles.^3^

CAA is associated with the dysfunction and loss of vascular smooth muscle cells (VSMCs),^3-5^ and patients with CAA have been observed to have a decline in stimulus-evoked cerebrovascular reactivity (CVR) as measured by blood oxygen level dependent functional MRI studies.^6^ Additionally, studies in pre-symptomatic individuals, defined as having no cognitive impairment or haemorrhagic lesions, with hereditary Dutch-type CAA have demonstrated declines in CVR,^7^ suggesting vascular dysfunction may precede symptomatic CAA.

In addition to their role in CVR, VSMCs also drive spontaneous slow oscillations in arterial diameter, termed vasomotion, at a frequency of ∼0.1 Hz.^8,9^ Prior work has suggested that vasomotion plays a key role in glymphatic clearance,^9,10^ with oscillations in vessel diameter increasing the mobility of the cerebrospinal fluid (CSF) in the perivascular space, promoting exchange of molecules in the brain tissue and CSF.^11^ Therefore, impairments in vasomotion could have implications for the accumulation of vascular amyloid-β in CAA.^12^ Notably, amyloid-β levels in CSF are lower in patients with CAA,^13^ indicating that clearance of waste from the brain tissue towards the CSF may be impaired. In addition to vasomotion, beat-to-beat pulsatility, the change in vessel diameter with each heartbeat, has been suggested as another driving force for glymphatic clearance.^14^ Beat-to-beat pulsatility has been shown to increase with age and cerebral small vessel disease, including CAA.^15^

We hypothesized that impairments in vasomotion would be observed with initial amyloid-β deposition, potentially contributing to a feed-forward mechanism in which reduced vasomotion leads to more deposition of amyloid-β, in turn leading to further declines in vasomotion. To study the progression of amyloid-β deposition, vascular dysfunction and haemorrhage in CAA, we used the APP23 mouse model of amyloidosis, a model known to exhibit severe CAA and spontaneous MRI-visible cerebral microbleeds.^16,17^ Longitudinal, unanesthetized *in vivo* 2-photon microscopy was performed in APP23 transgenic (Tg) mice and wildtype (WT) littermates from 7 to 14 months of age to track amyloid-β accumulation, arterial diameter, beat-to-beat pulsatility and spontaneous vasomotion at an individual vessel level. We then performed *in vivo* MRI in separate cohorts of Tg mice and WT littermates at later ages (12, 18 and 24 months), assessing cerebral microbleeds using a multi-gradient echo (MGE) sequence and global cerebrovascular function using pseudo-continuous arterial spin labelling (pCASL)-MRI, combined with a hypercapnic (CO_2_) stimulus. Lastly, immunohistochemistry was performed to assess vascular smooth muscle actin (SMA) coverage at 12, 18 and 24 months in post-mortem brain tissue of Tg mice and WT littermates.

## Materials and methods

### Animals

All experiments were approved by the animal care and use committee of Massachusetts General Hospital under protocol number 2018N000131. The study was compliant with the National Institutes of Health Guide for the Care and Use of Laboratory Animals and reported according to the ARRIVE guidelines.^18^ Some mice were acquired directly from the Jackson Laboratory, Bar Harbor, Maine (strain #030504); others were derived from in-house breeding (founded with strain #030504 mice from the Jackson Laboratory). Sample sizes were determined *a priori* based on prior laboratory experience with similar imaging data. Note, final sample sizes for MRI and immunohistochemistry groups were influenced by mouse availability at advanced ages. See further mouse details including housing information and which mice were bred in house in Supplementary Material—Detailed Methods. Different cohorts of mice were used for 2-photon microscopy and MRI.

#### 2-photon microscopy

Mice were imaged monthly longitudinally from ∼7 to 14 months of age as follows: 4 WT (1 female) and 4 Tg (2 female). The average age at first imaging session was 250 ± 30 days and 252 ± 29 days in WT and Tg mice respectively. An additional cohort was imaged at 15–16 months of age: 4 WT (1 female), 3 Tg (2 female). A third cohort of 15–16-month-old mice was used for experiments assessing the effects of isoflurane on vascular diameter: 3 WT (1 female), 3 Tg (1 female).

#### MRI

A cross-sectional study design was used with three groups of mice as follows: (i) 12 months: 6 WT (4 female), 5 Tg (4 female); (ii) 18 months: 10 WT (5 female), 10 Tg (4 female); and (iii) 24 months: 9 WT (3 female), 6 Tg (4 female).

Five mice (1 WT 12-month-old male, 1 WT 18-month-old male, 1 WT 18-month-old female, 2 Tg 18-month-old males) in the MRI cohort were excluded from the pCASL analysis, because of technical problems (see paragraph on MR image processing). One additional 18-month-old Tg female was excluded from the MRI cohort, due to detection of a large brain tumor-like lesion with MRI.

#### Immunohistochemistry

For histopathological analysis, the following mice were included: (i) 12 months: 3 WT (2 female), 3 Tg (3 female); (ii) 18 months: 7 WT (3 female), 6 Tg (3 female); and (iii) 24 months: 8 WT (3 female), 5 Tg (3 female).

### 2-photon microscopy

#### Surgical preparation

Cranial windows and headposts were implanted in mice as previously described.^9^ Briefly, mice were anesthetized with 1.5–2% isoflurane (in 100% O_2_), throughout surgical procedures. A dental drill was used to drill a 6 mm craniotomy overlying the bilateral somatosensory cortices, which was then replaced with an 8 mm glass coverslip, fixed in place with a mixture of dental cement and Krazy Glue. Dura was kept intact. Custom-designed stainless-steel headposts were fixed to the skull using C&B Metabond (Parkell). Buprenorphine (0.05 mg/kg) and Tylenol (300 mg/mL) were used for post-surgery analgesia.

#### Image acquisition

At least 4 weeks following each surgery, longitudinal 2-photon imaging was initiated. Prior to imaging, mice were habituated to awake imaging through at least two 5–15 min sessions of head fixation via headpost to a stereotactic frame on a circular treadmill. Mice were then imaged approximately monthly for up to 6 months. One day prior to each imaging session, 300 μL methoxy-XO4 solution (Glixx lab) [∼5 mg/kg, dissolved in 3% Cremophor®EL (Sigma) and phosphate-buffered saline (PBS)] was injected intraperitoneally to fluorescently label amyloid-β. On the day of imaging, mice were induced with 5% isoflurane and retro-orbital intravenous injections of 150 μL of fluorescein or Texas Red dextran (70 kDa) solution (12.5 mg/mL; Invitrogen) were performed. Mice were then allowed to wake-up over a 15-min period prior to imaging. During each imaging session, mice were fixed via headposts in a stereotactic frame and allowed to move freely on a circular treadmill.

Mice were imaged using a FluoView FV1000MPE 2-photon laser-scanning system (Olympus) mounted on a BX61QI microscope (Olympus), equipped with a 25× (numerical aperture = 1.05) dipping water immersion objective (Olympus). A MaiTai® DeepSee™ Ti:Sapphire mode-locked laser (Spectra-Physics) generated 2-photon excitation at 800 nm. External detectors containing three photomultiplier tubes (Hamamatsu) collected emitted light at 420–460 nm (blue), 495–540 nm (green) and 575–630 nm (red). Laser power was maintained at 10–20% in all imaging sessions. Each field of view (FOV) was imaged approximately monthly, unless precluded by clouding of the window. See imaging parameters in Supplementary Material—Detailed Methods. Longitudinal 2-photon imaging of mice concluded after 6 months of imaging, if clouding of the window precluded further imaging (*n* = 2, each after 3 months of imaging), or with death of the mouse (*n* = 1, after 4 months of imaging).

#### Image processing

2-photon images were analyzed in Fiji and MATLAB using in-house developed scripts. To assess spontaneous vasomotion, vascular diameter was assessed in arteriolar segments in 5 min timeseries data using the dextran channel data (either green or red channel); the methoxy-XO4 channel (blue) was subtracted because of bleed-through of the methoxy-XO4 signal in the other channels. Fast-Fourier transforms were performed over the full 5-min time-series and the peak amplitude in the frequency domain between 0.04 and 0.13 Hz was calculated for each segment. To avoid pseudoreplication, an average ‘vasomotion peak’ was calculated for each FOV in each imaging session by averaging the vasomotion peaks across ∼2–4 arteriolar segments within the FOV. For details regarding assessments of % CAA coverage, baseline arteriolar diameter, red blood cell (RBC) velocity and beat-to-beat pulsatility, see Supplementary Material—Detailed Methods.

### MRI

#### Image acquisition

For MRI acquisition, mice were anesthetized with a low isoflurane protocol previously described in detail.^19^ In short, anaesthesia was induced with 2% isoflurane and maintained at 1.1% in oxygen-enriched air (30% O_2_). The mice were head-fixed. Breathing rates were monitored using a pressure-sensitive pad placed below the animal. Temperature was maintained at 36.5°C using a feedback-controlled air heat pump (Kent Scientific). A 9.4 T MRI scanner with Paravision 6.0.1 software (Bruker, Ettlingen, Germany) was used with a head phased array receive coil and whole-body volume transmit coil. See scan details in Supplementary Material—Detailed Methods.

In a subset of 18-month-old mice from the MRI cohort (3 WT and 3 Tg), 1 week before the MRI scan, mice were placed in the same cradle as used during MRI, and underwent the anaesthesia protocol described above, including 10% CO_2_ stimulation. Instead of MRI scanning, the right dorsal flank was shaved, and transcutaneous (tc)-pCO_2_ was measured using a skin probe coupled to a blood gas analyzer (CombiM54, Radiometer America, CA).

#### Image processing

The pCASL image analysis pipeline has been described in detail previously.^19^ In short, consecutive EPIs from individual scans were first spatially aligned. Cerebral blood flow (CBF) maps were generated for each label and control pair, using MATLAB-based open-source software.^20^ CBF was expressed in mL/100 g/min, derived from the label and control signal difference using Buxton's perfusion model^21^ as follows:


$$\mathrm{CBF}=\frac{\lambda \times \Delta M\times e^{\left(\frac{\mathrm{PLD}}{T_{1b}}\right)}\;}{2\times \alpha \times T_{1t}\times M_{0t}\times \left(1-\;e^{\left(\frac{-\tau }{T_{1t}}\right)}\right)}$$

Here, *λ* is the blood–brain partition coefficient (0.9 mL/g^22^), Δ*M* is the pCASL label and control signal difference and *T*_1*b*_ is the longitudinal relaxation time of blood, taken to be 2430 ms at 9.4 T.^23^ The labelling efficiency (*α*) was assumed to be 0.80, which was the median *α* of all WT animals (Supplementary Fig. 6) and is in correspondence with literature.^24^ ROIs were drawn by hand on the reference T_2_-weighted RARE scan to extract regional CBF values. CBF time-profiles were smoothed by removing outliers (exclusion of values higher or lower than two times the standard deviation) and applying a three-point moving average. CVR time-profiles were derived by normalising all CBF values to the average CBF value between minute 1 and 5. Average CVR values were derived by computing the relative increase in CBF during minute 6–10 over the CBF during minute 1–5. See further image processing details in Supplementary Material—Detailed Methods.

Five mice were excluded from the pCASL analysis, because of ghosting artefacts in the EPIs. Because the ghosts were variable in signal intensity, the brain signal varied randomly over time, resulting in large subtraction errors when deriving the perfusion signal. The ghosts likely appeared in these five datasets due to insufficient head fixation, resulting in respiration-related movement.

The MGE scans were inspected in MATLAB by two blinded observers (M.G.K. and M.L.B), to determine the number of cortical microbleeds, defined as hypointense round or ovoid lesions. The intraclass correlation coefficient was excellent (0.833). Final microbleed counts were determined at a consensus meeting.

### Immunohistochemistry

After completion of MRI scans, mice were euthanized on the same day through CO_2_ asphyxiation and transcardially perfused with 20 mL PBS. When possible, 2-photon imaged mice were also euthanized and transcardially perfused within 1 week of the last 2-photon imaging session. Brains were extracted, fixed in 4% paraformaldehyde with 15% glycerol in PBS for several months, and then embedded in paraffin in a coronal orientation. Serial sections of 6 μm thickness were cut with a microtome.

Serial sections from a subset of mice (see *Animals* section) underwent immunohistochemistry against amyloid-β and SMA. Staining details are provided in Supplementary Material—Detailed Methods. Sections were imaged using the Hamamatsu NanoZoomer Digital Pathology (NDP)-HT scanner (C9600-12, Hamamatsu Photonics K.K., Japan) at 20 × magnification. The viewing platform NDP.View (v2.6.13) was used to analyze the digital sections.

A trained rater (C.A.A.) selected all pial arterioles from one amyloid-β stained coronal section at approximately the level of the somatosensory cortex from each mouse (identified using the Allen Mouse Brain Atlas).^25^ Vessels were rated on the Vonsattel grading scale^3^ as follows: Vonsattel Grade 0 (no amyloid-β), Vonsattel Grade 1 (patchy amyloid-β deposition), and Vonsattel Grade 2 (circumferential amyloid-β). At least 1 week after vessel selection, blinded to the mouse age, genotype, and Vonsattel scores, the rater scored each selected vessel on adjacent SMA stains on the following SMA scale (modified from^5^): 0 (no SMA), 1 (incomplete SMA coverage), 2 (complete SMA coverage). Arterioles in 2 randomly selected amyloid-β stains (both Tg) and 2 SMA stains were rated by a second rater (M.G.K.) for both Vonsattel grades and SMA scores. Inter-rater reliability was excellent for both Vonsattel grades (kappa = 0.96) and SMA scores (kappa = 0.87). An average SMA score per Vonsattel grade was then generated for each mouse.

### Statistics

Statistical analyses were performed in R, Prism v10, and MATLAB. Linear mixed effects (LME) models were fitted using the ‘lme4’ package version 1.1–26 in R to assess changes in vascular amyloid-β, vessel diameter, RBC velocity, vasomotion and beat-to-beat pulsatility. All models tested for heteroskedasticity, and log transforms were used as needed. In all models, subject (i.e. mouse ID) and arteriole were treated as random effects (random intercept and/or random slope terms as specified in each table). Fixed effects used in each model are detailed in the Results section, Table 1, and Supplementary Tables 1–6. Model fits were compared by evaluating the Akaike information criterion and Bayesian information criterion for each model; lower values indicated a better fit.

**Table 1:** Vasomotion models

		Null Model	Model 1	Model 1b	Model 2	Model 2b	Model 3	Model 4
CAA coverage (%)	*P*-value		***P* = 3.3E−6**	***P* = 6.6E−6**	***P* = 1.5E−4**	***P* = 2.1E−4**	***P* = 6.8E−4**	*P* = 0.95
	estimate		−3.8E−03	−3.8E−03	−3.4E−03	−3.4E−03	−3.1E−03	6.7E−05
	95% CI		[−5.3E−3 −2.4E−3]	[−5.3E−3 −2.3E−3]	[−5.1E−3 −1.6E−3]	[−5.1E−3 1.8E−3]	[−4.9E−3 −1.3E−3]	[−2.2E−3 2.3E−3]
Age	*P*-value				*P* = 0.30	*P* = 0.30	*P* = 0.23	*P* = 0.42
	estimate				−1.1E−02	−1.1E−02	−1.2E−02	8.9E−03
	95% CI				[−3.1E−2 9.5E−3]	[−3.1E−2 9.5E−3]	[−3.3E−2 8E−3]	[−0.013 0.031]
Genotype (Tg)	*P*-value						*P* = 0.40	***P* = 2.2E−4**
	estimate						−4.4E−02	8.7E−01
	95% CI						[−0.15 6.4E−2]	[0.42 1.33]
Age × Genotype (Tg)	*P*-value							***P* = 7.3E−5**
	estimate							−9.7E−02
	95% CI							[−0.14 −0.050]
Log likelihood		26.0	38.7	38.7	39.3	39.3	39.7	**47.5**
AIC		−43.9	−67.5	−65.5	−66.6	−64.6	−65.3	**−79.0**
BIC		−32.3	−53.0	−48.1	−49.1	−44.2	−45.0	**−55.8**
Total *R*^2^		0.21	0.38	0.38	0.37	0.30	0.37	**0.45**

Null Model = log10(Vasomotion) ∼ 1 + (1|Mouse) + (1|FOV).

Model 1 = log10(Vasomotion) ∼ Abeta + (1|Mouse) + (1|FOV).

Model 1b = log10(Vasomotion) ∼ Abeta + (1|Mouse) + (Age||FOV).

Model 2 = log10(Vasomotion) ∼ Abeta + Age + (1|Mouse) + (1|FOV).

Model 2b = log10(Vasomotion) ∼ Abeta + Age + (1|Mouse) + (Age||FOV).

Model 3 = log10(Vasomotion) ∼ Abeta + Age + Genotype + (1|Mouse) + (1|FOV).

Model 4 = log10(Vasomotion) ∼ Abeta + Age × Genotype + (1|Mouse) + (1|FOV).

Results for the fixed effects estimates of LME models developed with arteriolar vasomotion peak as the dependent variable. In all models, mouse and vessel were treated as random effects. Models with random intercept terms (Model 1, Model 2, Model 3 and Model 4) and random intercept and random slope terms (Model 1b and Model 2b) are shown. Note, random intercept terms were used alone in Models 3 and 4 as the addition of random slope terms did not improve the performance of Models 1 and 2. Significant *P*-values in bold for each model. Model 4 had the best fit, and this model included all fixed effects, with significant contributions of genotype and age × genotype interactions (*n* = 135 measurements over 4 Tg and 4 WT mice, statistical measures in bold).

Further details regarding statistical analyses are included in Supplementary Material—Detailed Methods.

## Results

### Vascular amyloid-β starts to accumulate in pial arterioles at ∼8–10 months of age in APP23 Tg mice

**Figure 1:**
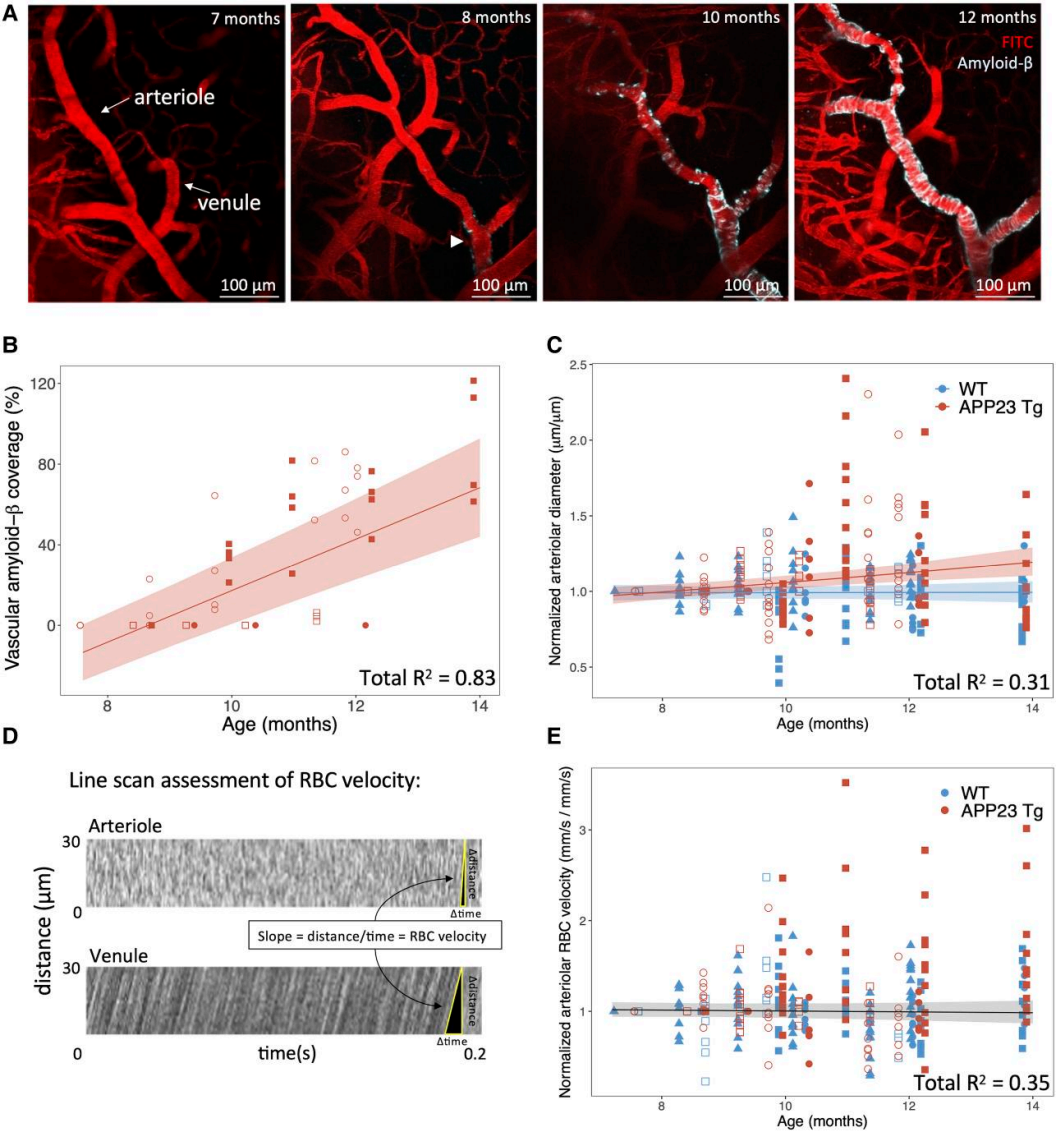
**Vascular amyloid-β deposition starts to accumulate in APP23 Tg mice around 8–10 months of age and increasing age in APP23 Tg mice is associated with an increase in baseline vessel diameter.** (**A**) *In vivo* 2-photon images of the same arteriole and venule in a representative APP23 Tg mouse imaged from 7 to 12 months of age using intravascular fluorescein isothiocyanate-labeled 70 kDa dextran (pseudocolored red here for optimal contrast). Amyloid-β was first observed along the arteriole (arrowhead) at 8 months (visualized with methoxy-XO4 in cyan). (**B**) % vascular amyloid-β coverage of each pial arteriole in APP23 Tg mice versus age. Each mouse is depicted with a different symbol (*n* = 4 Tg mice, 4 pial arterioles per mouse). Line represents LME model with age as the predictor variable, and mouse and vessel as random slopes (shaded areas represent the 95% confidence interval of the model's prediction, Model 1b, see Supplementary Table 1). % vascular amyloid-β increases with age (*P* = 1.8E−9). (**C**) Normalized arteriolar diameter versus age (each arteriolar segment diameter normalized to initial imaging session). Each mouse is depicted with a different symbol (*n* = 4 Tg, 4 WT mice, 355 measurements across mice). Line represents LME model with best fit including mouse and vessel as random slopes, and age, genotype, and age × genotype interactions as fixed effects (shaded areas represent the 95% confidence interval of the model's prediction, Model 3, see Supplementary Table 2). No change in vessel diameter with age in WT mice, increases in vessel diameter with age in Tg mice (*P* = 0.036), with significant interaction between age × genotype (*P* = 7.1E−3). (**D**) Representative examples of arteriole and venule (for reference) RBC velocity calculations from line scan data. (**E**) Normalized arteriolar RBC velocity plotted versus age (each arteriole segment normalized to first imaging session). Line represents LME model including mouse and vessel as random slopes, and age as a fixed effect (shaded areas represent the 95% confidence interval of the model's prediction, Model 1, see Supplementary Table 3). No significant association with age was observed in this model. Open symbols = female, closed symbols = male (**B**, **C**, **E**).

CAA was first observed in pial arterioles in Tg mice between ∼8 and 10 months of age in 3/4 Tg mice imaged (Fig. 1). To assess the relationship between age and vascular amyloid-β (i.e. CAA) coverage, LME models were applied with age as the predictor variable, mouse and vessel as either random intercepts or random slopes, and % CAA coverage as the dependent variable. % CAA coverage increased significantly with age (*n* = 62 measurements across 4 Tg mice, *P* = 1.8E−9) (Fig. 1B). The model including age as a fixed effect and mouse and vessel as random slopes explained 83% of the variance in % CAA coverage (Model 1b, comparison between null and full model: *c*^2^ = 76.8, *P* = 2.2E−16). See Supplementary Table 1 for additional model details. Note, one mouse did not develop CAA within the captured FOVs over the course of imaging. This mouse was re-genotyped confirming it was Tg and therefore was included in all analyses.

### Pial arteriolar diameter increases with age in APP23 Tg mice, while RBC velocity remains unchanged

Pial arteriolar diameter was observed to increase significantly with age in Tg mice, while remaining unchanged in WT littermates (Fig. 1C). LMEs were applied with mouse and vessel as random effects (random slopes), and age, genotype, and age × genotype interactions as fixed effects, and normalized diameter as the dependent variable. The model with the best fit (Model 3) included all three fixed effects, demonstrating an increase in normalized arteriolar diameter with Tg genotype (*P* = 0.036) and age × genotype interaction (*P* = 7.1E−3) (*n* = 355 vessel measurements across 4 Tg and 4 WT mice). This model explained 31% of the variance in arteriolar diameter (comparison between null and full model: *c*^2^ = 32.8, *P* = 3.5E−7). See Supplementary Table 2 for additional model details.

Arteriolar RBC velocity was found to be unchanged with age in both Tg and WT mice (Fig. 1D). LMEs were built as described for arteriolar diameter, and no significant relationships between age and/or genotype and RBC velocity were observed (*n* = 334 measurements across 4 Tg and 4 WT mice). See Supplementary Table 3 for additional model details.

Note, there were no significant differences in baseline arteriolar diameter or baseline arteriolar RBC velocity between WT and Tg mice (students *t*-test, Supplementary Fig. 1).

### Spontaneous vasomotion declines with age in APP23 Tg mice

**Figure 2:**
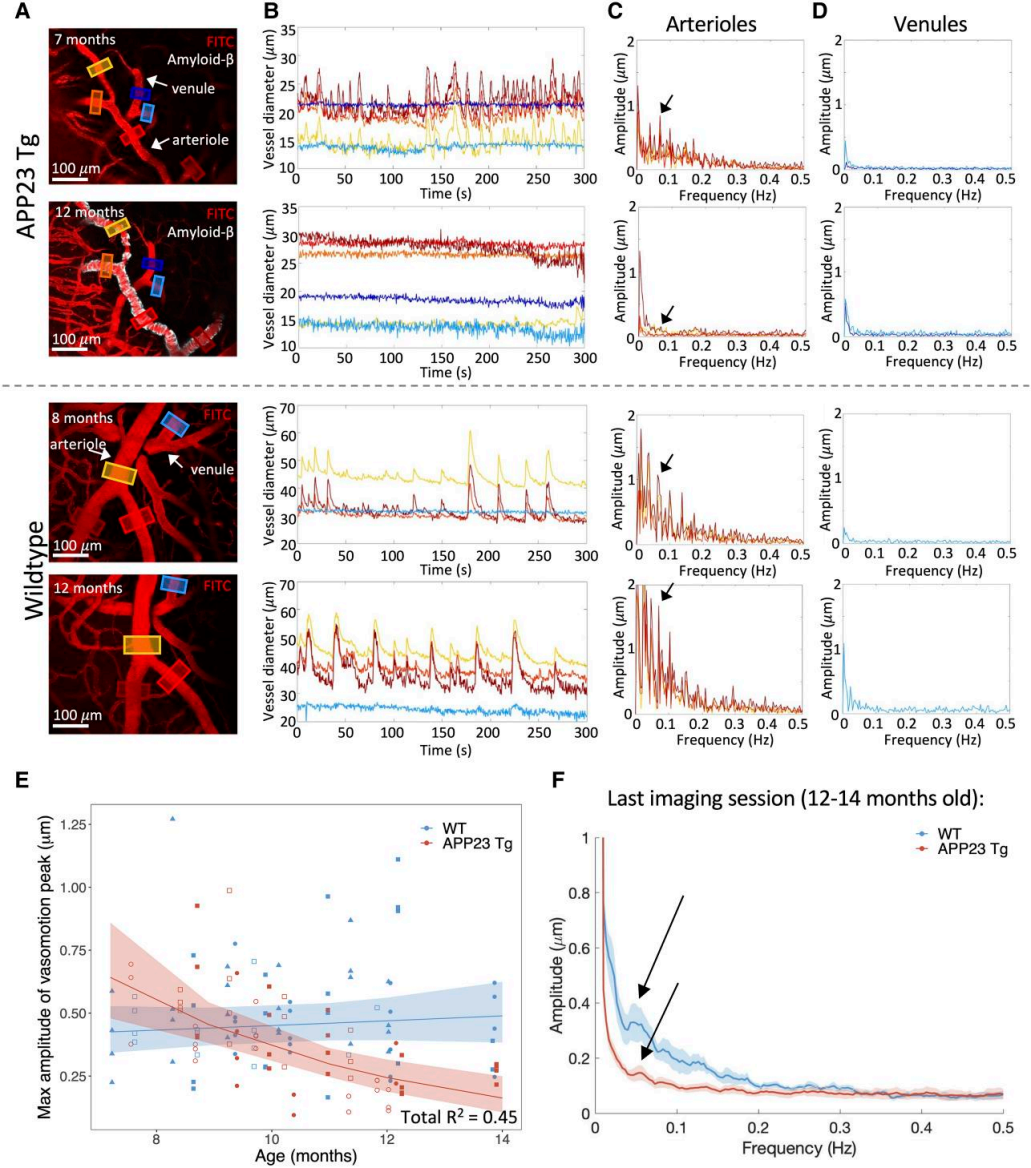
**Spontaneous vasomotion decreases significantly with age in APP23 Tg mice.** (**A**) Representative examples of APP23 Tg and WT mice at 7 and 12, and 8 and 12 months old respectively [vasculature visualized with intravascular fluorescein isothiocyanate-labeled 70 kDa dextran (pseudocolored red), amyloid-β visualized with methoxy-XO4 (cyan)]. (**B**) Vessel diameter time courses from ROIs depicted in (**A**). Arterioles are observed to have spontaneous fluctuations in vessel diameter, while venules do not notably change in diameter. These fluctuations in vessel diameter are no longer present in Tg mice at 12 months of age, while remaining intact in WT mice at 12 months. (**C**) and (**D**) Fast-Fourier transform of time courses shown in (**B**) for arterioles (**C**) and venules (**D**). Arrows point to the peak detected amplitude between 0.04 and 0.13 Hz, corresponding to the ‘vasomotion peak’ frequency. (**E**) Graph of the max amplitude of the vasomotion peak in arterioles versus age (*n* = 4 Tg, 4 WT mice, 135 total measurements). Each mouse is represented by a different symbol. Open symbols = female, closed symbols = male. LME with the best fit shown, including mouse and vessel as random effects (intercepts), and % CAA coverage, age, genotype, and age × genotype interactions as fixed effects (shaded areas represent the 95% confidence interval of the model's prediction, Model 4, see Table 1). Both genotype and age × genotype interactions were significant predictors (*P* = 2.2E−4 and *P* = 7.3E−5 respectively). (**F**) Averaged fast-Fourier transforms of time courses from the last imaging sessions from each mouse (*n* = 4 WT, 4 Tg mice). Mean time courses ± SEM (shaded) shown. Arrows point to vasomotion peaks in APP23 Tg and WT mice.

Spontaneous vasomotion in pial arterioles was observed to decline with age in Tg mice, while remaining unchanged in WT littermates (Fig. 2). LMEs were applied with arteriolar vasomotion peak as the dependent variable, mouse and vessel as random effects (random intercept ± random slope as specified in Table 1), and % CAA coverage, age, genotype and age × genotype interactions as fixed effects. Note, random intercept terms were used in Models 3 and 4 as random slope terms did not improve the performance of Models 1 and 2. The model with the best fit (Model 4) included all fixed effects, with significant contributions of genotype (*P* = 2.2E−4) and age × genotype interactions (*P* = 7.3E−5) (*n* = 135 measurements over 4 Tg and 4 WT mice). Notably, in this model, % CAA coverage in the imaged FOVs did not have a significant effect (but did have a significant effect in models not including an age × genotype interaction), suggesting that age × genotype interactions may be better predictors of vasomotion decline than local % CAA coverage. This model explained 45% of the variance in the data and fit significantly better than all models tested (all model details in Table 1, comparison between null and full model: *c*^2^ = 43.1, *P* = 9.7E−9). No difference was observed in the peak vasomotion frequency between Tg and WT mice, and there was no shift in the peak frequency over the imaging period (Supplementary Fig. 2).

To confirm that these changes in vasomotion were not secondary to differential effects of longitudinal cranial windows on Tg mice as compared with WT mice, a separate cohort of mice ∼15–16 months of age were imaged awake 1 month following cranial window surgery (*n* = 4 WT, 3 Tg; Supplementary Fig. 3). Data from these mice was compared with 7–9-month-old and 12–14-month-old timepoints from our longitudinal cohort using a two-way ANOVA. We observed a significant age × genotype interaction (*P* = 4.7E−3, *F* = 7.5) and significantly lower vasomotion associated with the Tg genotype (*P* = 3.4E−3, *F* = 11.6). Pre-selected comparisons were performed using Šídák correction, demonstrating no significant differences between 7–9-month-old WT and 7–9-month-old Tg mice or 7–9-month-old WT and 15–16-month-old WT mice. Significant differences were observed between 12–14-month-old WT and 12–14-month-old Tg mice (*P* = 5.2E−3), 15–16-month-old WT and 15–16-month-old Tg mice (*P* = 0.039) and 7–9-month-old Tg and 15–16-month-old Tg mice (*P* = 8.2E−3).

### Beat-to-beat pulsatility increases with age in both APP23 Tg mice and WT littermates

**Figure 3:**
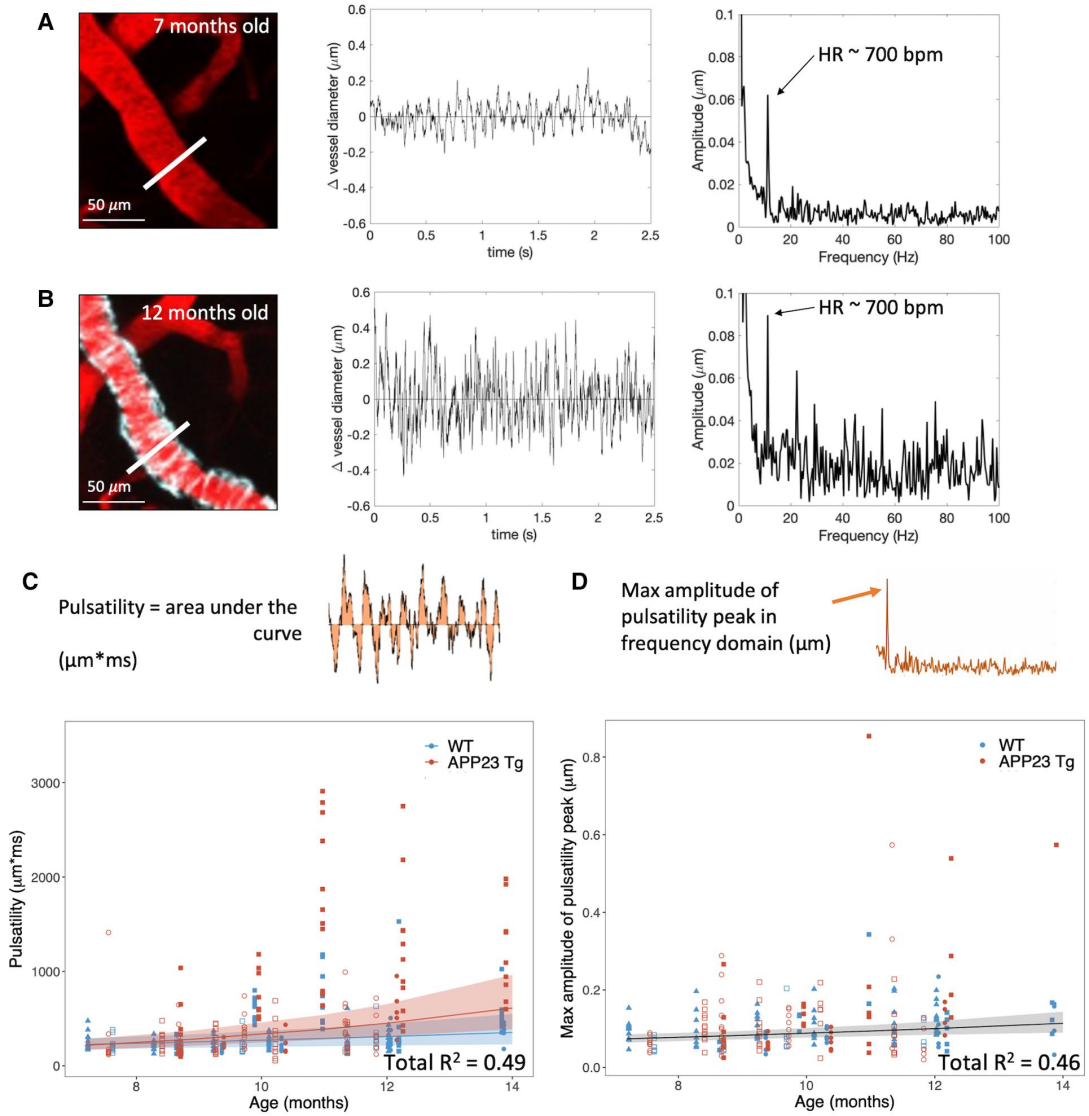
**Beat-to-beat pulsatility increases with age in both APP23 Tg and WT mice.** (**A**) Representative arteriole in a 7-month-old APP23 Tg mouse visualized using intravascular FITC-labeled 70 kDa dextran (pseudocolored in red) and *in vivo* 2-photon microscopy (left), vessel diameter time course from rapid line scan (centre), and fast-Fourier transform of this time course (right). Arrow pointing to peak frequency, representing heart rate (HR) in beats per minute (bpm). (**B**) Same measurements from the same arteriolar segment ∼5 months later (amyloid-β visualized with methoxy-XO4 in cyan). (**C**) Pulsatility measurements (calculated as AUC, diagram shown) versus age (*n* = 4 Tg, 4 WT mice, 355 measurements total). LME with best fit shown, including mouse and vessel as random effects (slopes), and diameter, age, genotype, and age × genotype interactions as fixed effects. Increases in pulsatility associated with increasing vessel diameter (*P* = 5.0E−10), age (*P* = 1.2E−2), Tg genotype (*P* = 0.043), and age × genotype (Tg) interactions (*P* = 0.026) (shaded areas represent the 95% confidence interval of the model's prediction, Model 3b, see Supplementary Table 4). Each mouse is represented by a different symbol. (**D**) Pulsatility measurements (calculated as the peak amplitude in the frequency domain, diagram shown) versus age (*n* = 4 Tg, 4 WT mice, 242 measurements total). LME with best fit shown, including mouse and vessel as random effects (slopes), and age as a fixed effect, demonstrating increases in pulsatility with age (*P* = 1.9E−3) (shaded areas represent the 95% confidence interval of the model's prediction, Model 1b, see Supplementary Table 5). Open symbols = female, closed symbols = male (**C**, **D**).

To assess beat-to-beat pulsatility, arteriolar diameter was measured at high temporal resolution using bidirectional line scans (Fig. 3). Pulsatility was calculated as both the absolute value of the area under the curve (AUC) of the normalized diameter tracing after detrending (Fig. 3C) as well as the peak amplitude of the diameter tracing in the frequency domain (Fig. 3D, corresponding to heart rate). Both metrics were observed to increase significantly with age in Tg mice and WT littermates. For AUC comparisons, an LME was built with mouse and vessel as random effects (either as random intercepts or random slopes as specified in Supplementary Table 4), and diameter, age, genotype, and age × genotype interactions as fixed effects (*n* = 355 measurements across 4 Tg and 4 WT mice). The model with the best fit (Model 3b, see Supplementary Table 4) included mouse and vessel as random slopes and demonstrated increases in pulsatility with vessel diameter (*P* = 5.0E−10), age (*P* = 0.012), Tg genotype (*P* = 0.043), and age × genotype interactions (*P* = 0.026), indicating that pulsatility increases in both Tg and WT mice with age, and that this effect is more prominent in Tg mice. This model explained 49% of the variance in the pulsatility (comparison between null and full model: *c*^2^ = 115.8, *P* < 2.2E−16).

To minimize potential effects of noise in the measurements, these data were also analyzed in the frequency domain with a fast-Fourier transform, assessing the amplitude peak at the heart rate frequency. Measurements were excluded (*n* = 113 measurements across 8 mice) from this analysis if the calculated heart rate peak was >50% different from the heart rate of the animal (as determined by RBC velocity tracings), as this was suggestive of noise in the data. Again, LMEs were built with the same parameters as above (*n* = 242 measurements across 4 Tg and 4 WT mice). The model with the best fit (Model 1b, see Supplementary Table 5) included mouse and vessel as random slopes, and age (and not genotype or age × genotype interaction) as a fixed effect, demonstrating increases with age in both groups (*P* = 1.9E−3). This model explained 46% of the variance in the pulsatility (comparison between null and full model: *c*^2^ = 19.7 *P* = 9.3E–6). These findings confirm that pulsatility increases with age in Tg and WT mice but suggest that this effect may not be enhanced by Tg genotype.

Note, no significant differences in baseline heart rate were observed between Tg and WT mice, as measured from velocity line scans (Supplementary Fig. 1). Additionally, no significant changes in heart rate were observed with increasing age in either Tg or WT mice (Supplementary Fig. 4 and Table 6).

### Vascular amyloid-β accumulation and declines in vasomotion typically precede diameter and pulsatility changes

Vascular amyloid-β (% coverage) and changes in vasomotion, arteriolar diameter and pulsatility (% change from baseline levels) were compared across individual FOVs from each Tg mouse in which all parameters were measured in ≥3 imaging sessions (total of 9 FOVs across 4 mice; Supplementary Fig. 5). Initial amyloid-β deposition and a > 25% decrease in vasomotion were observed to precede or occur at the same time point as >25% changes in diameter and pulsatility in 7/9 FOVs (Supplementary Fig. 5A). A summary of the sequence of events observed in each mouse is shown in Supplementary Fig. 5B and a schematic of the generally observed sequence of events of vascular structure/function changes is shown in Supplementary Fig. 5C.

### Baseline CBF is decreased in APP23 Tg mice at 24 months of age

**Figure 4:**
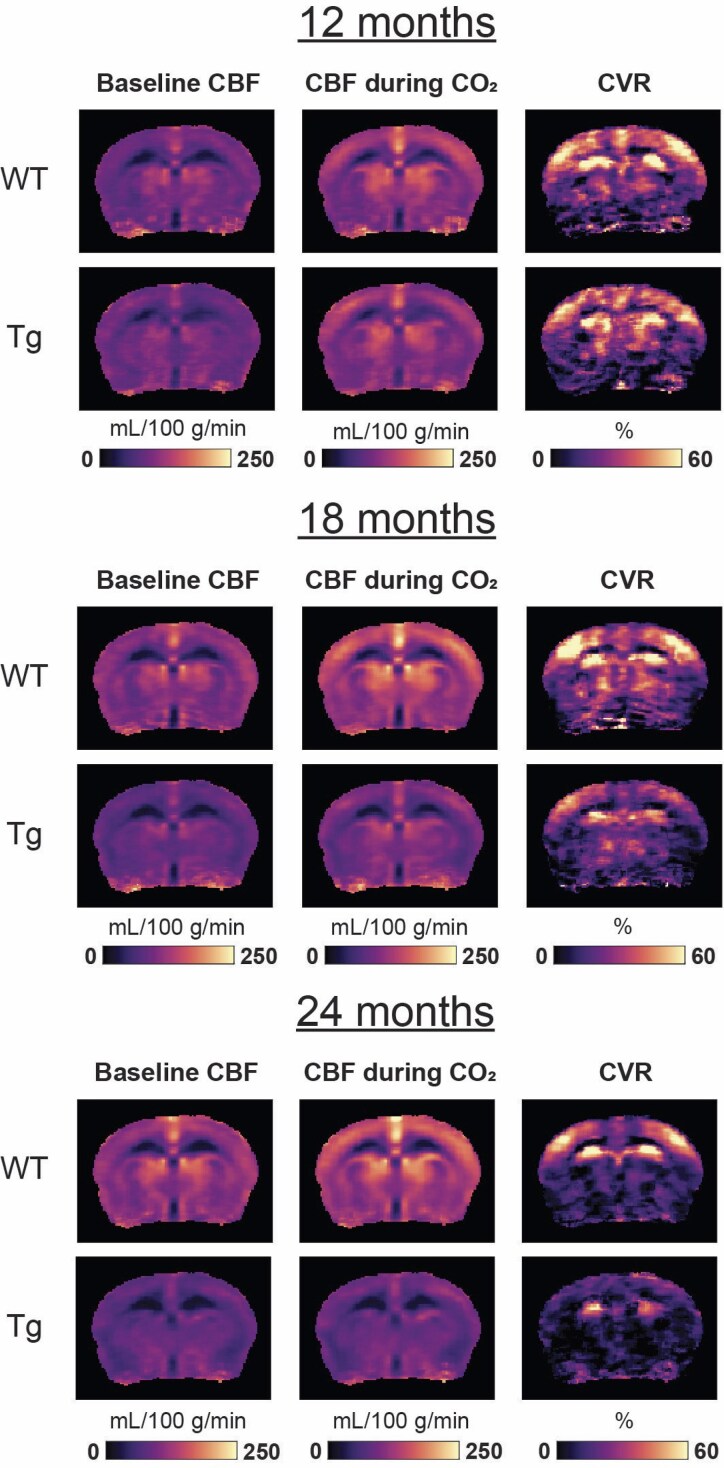
**MRI maps of CBF and CVR.** The rows display maps averaged per genotype (WT or APP23 Tg) and per age group (12, 18 or 24 months old), with each group consisting of *n* = 5–9 mice. On the columns, the maps display the baseline CBF, CBF during CO_2_, and CVR (relative CBF change during CO_2_). Note the similarity of the WT and APP23 Tg maps at 12 months of age, and the visibly lower CBF and CVR at 24 months of age. Also note that only the middle brain slice is shown, the results of the other 4 slices are shown in Supplementary Fig. 6.

**Figure 5:**
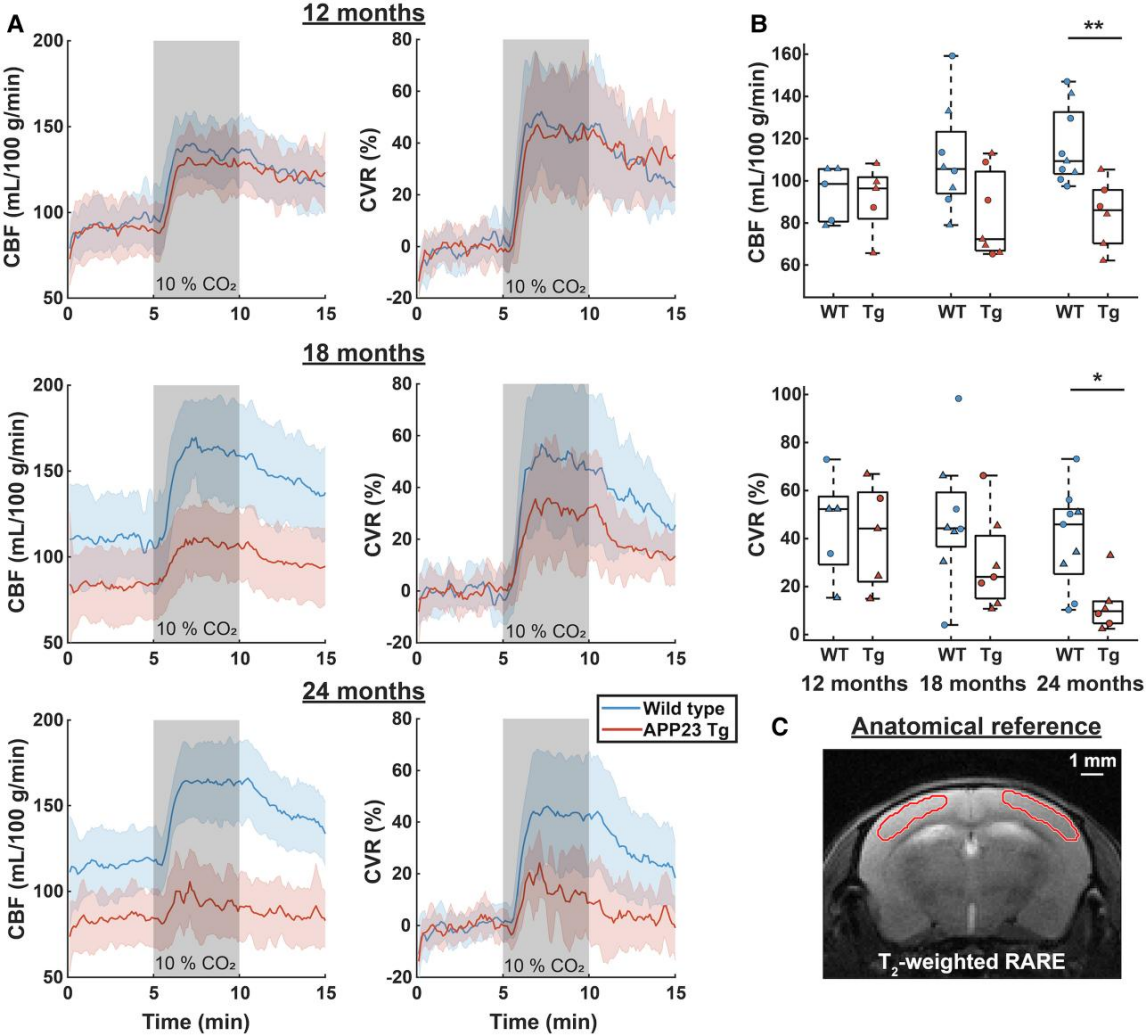
**CBF and CVR values in the somatosensory cortex.** In the left column of (**A**), the average CBF time-profiles ±SD are shown, per genotype WT in blue, APP23 transgenic [Tg] in red) and per age group, with each group consisting of *n* = 5–9 mice. The right column displays CVR time-profiles, i.e. baseline-corrected CBF time-profiles. In (**B**), boxplots are shown per genotype and age-group, where every point represents the average CBF (top) or CVR (bottom) per mouse. Circles represent male and triangles represent female mice. CBF was significantly lower in the 24-month-old APP23 Tg mice when compared with WT mice (*Z* = 2.77, *P* = 0.0056, Mann–Whitney U test). CVR was also significantly lower in the 24-month-old APP23 Tg mice (*Z* = 2.42, *P* = 0.016, Mann–Whitney U test). In (**C**), the red outline displays the somatosensory cortical region that was analyzed, overlaid on the reference T2-weighted Rapid Acquisition with Relaxation Enhancement (RARE) scan. * *P* < 0.05, ** *P* < 0.01.

Separate cohorts of mice were imaged with *in vivo* 9.4 T MRI to capture CBF and CVR in Tg and WT mice at advanced ages. Figure 4 displays averaged CBF and CVR maps across the age groups (12, 18 and 24 months) for Tg and WT mice in a single brain slice. The results for the other four brain slices are shown in Supplementary Fig. 6. Globally decreased baseline CBF was observed in the maps of 18- and 24-month-old (but not 12-month-old) Tg as compared to WT mice. Figure 5A shows the absolute and baseline-corrected group-averaged CBF time-profiles (mean ± SD) retrieved in the somatosensory cortex. Baseline CBF was significantly lower in 24-month-old Tg (86 mL/100 g/min) as compared to WT mice (109 mL/100 g/min, *Z* = 2.77, *P* = 5.6E−3). No significant reduction was found in the 12- or 18-month-old cohorts, but there was a trend towards a lower median CBF at 18 months (72 mL/100 g/min in Tg versus 106 mL/100 g/min in WT mice, *Z* = 1.91, *P* = 0.056).

### CVR in response to a CO_2_ challenge is decreased in APP23 Tg mice by 24 months of age

The CVR maps demonstrate differences in CVR between WT and Tg groups predominantly in the somatosensory cortex in the 18- and 24-month-old cohorts (but not the 12-month-old) (Fig. 4). In the 24-month-old Tg mice, CVR was significantly lower as compared to WT mice (10% in Tg versus 47% in WT, *Z* = 2.42, *P* = 0.016), and non-significant trends for lower CVR in the 18-month-old Tg mice were also observed (25% in Tg versus 45% in WT, *Z* = 1.22, *P* = 0.22) (Fig. 5).

Visual inspection of angiograms acquired with TOF did not demonstrate abnormalities in the larger arteries of Tg mice (Supplementary Fig. 7). Furthermore, no differences were found in the labelling efficiency between the Tg and WT mice (Supplementary Fig. 8), nor in the magnitude of change in transcutaneous pCO_2_ following the CO_2_ challenge (Supplementary Fig. 9).

### Exploratory experiment suggests that vasodilatory response to isoflurane is reduced in APP23 Tg mice

Given that the 2-photon experiments demonstrated an increase in baseline pial arteriolar diameter in Tg mice with age (and no change in RBC velocity), one might have expected an increase rather than a decrease in CBF in the Tg mice as compared to WT mice. A potential explanation for the apparent discrepancy between these results is a differential response to the light isoflurane anaesthesia (1.1%) used in the MRI experiments. Note, all 2-photon imaging was performed unanesthetized. We hypothesized that arterioles in Tg mice would not dilate significantly in response to isoflurane, potentially leading to relatively lower pCASL-based baseline CBF as compared to WT mice. We performed an exploratory 2-photon experiment in 15–16-month-old Tg and WT mice (*n* = 3 WT, 3 Tg) in which we systematically increased the level of anaesthesia from unanesthetized to 0.5%, 1.1%, 1.5% and 2% isoflurane. As expected, we observed large increases in vessel diameter in 2/3 WT mice. In comparison, we observed smaller increases in vessel diameter in 2/3 of the Tg mice (Supplementary Fig. 10). However, these were trends, and no significant differences were observed between Tg and WT groups (student's *t*-test). Given these findings, it remains unclear whether the decrease in baseline CBF observed with MRI would also be observed in the awake condition, or whether it reflects an impaired vasodilatory response to isoflurane.

### More than half of the APP23 Tg mice developed cortical microbleeds by 18 months of age

**Figure 6:**
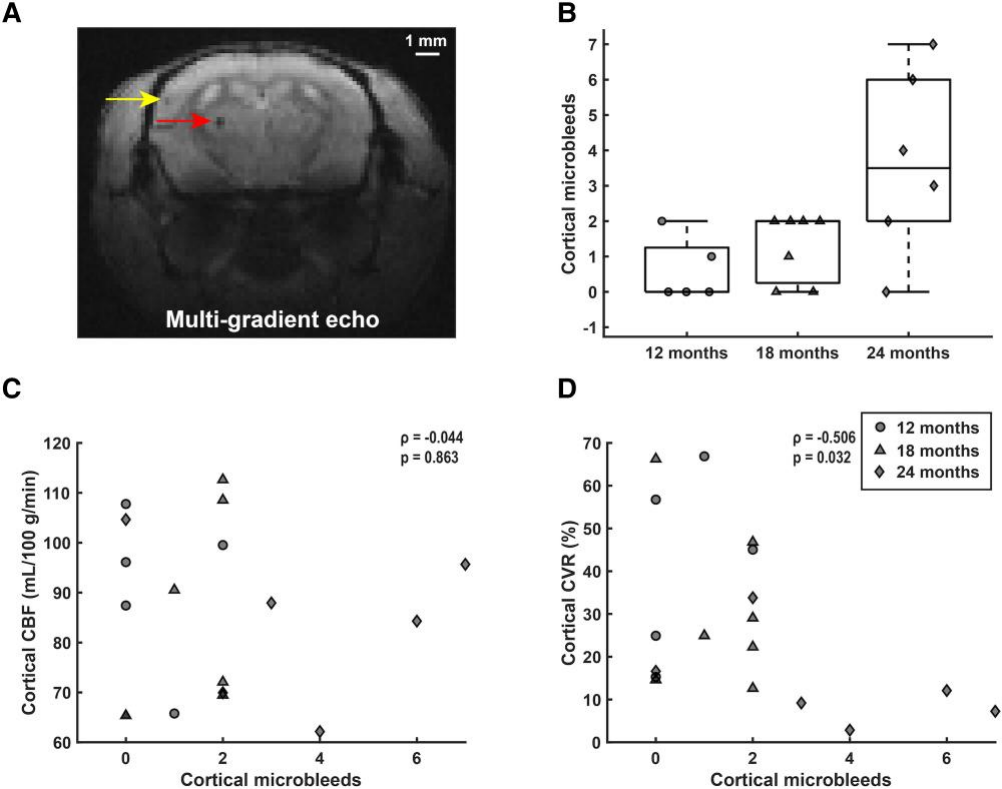
**Microbleed quantification and correlation with CBF and CVR in APP23 transgenic (tg) mice.** Microbleeds were scored in APP23 Tg mice by counting hypointense round or ovoid lesions in the cortex on MGE scans, as illustrated by the yellow arrow in (**A**). Note that sub-cortical hypointensities (red arrow) were not scored. (**B**) shows the number of cortical microbleeds per age-group, with *n* = 5 [4 female], *n* = 7 [4 female] and *n* = 6 [4 female] for the 12-, 18- and 24-months groups respectively. In (**C**), the number of microbleeds over all age-groups is plotted against cortical CBF, which did not reveal a correlation (Spearman's *ρ* = −0.044, *P* = 0.863). Circles, triangles and diamonds represent 12, 18 and 24-month-old mice respectively. In (**D**), the number of cortical microbleeds over all age-groups is plotted against cortical CVR, which revealed a significant correlation (Spearman's *ρ* = −0.506, *P* = 0.032).

To assess if the reductions in CBF and CVR in Tg mice were correlated with the occurrence of MRI-visible cortical microbleeds, MGE sequences were used to rate the number of cortical microbleeds in Tg mice (Fig. 6A). At 12 months of age, microbleeds were observed in 2/5 Tg mice (range 0–2). At 18 months of age, microbleeds were observed in 5/7 Tg mice (range 0–2). The highest number of microbleeds was observed at 24 months of age (in 5/6 mice, range 0–7). Across all three cohorts, the number of microbleeds inversely correlated with CVR (Spearman's *ρ* = −0.506, *P* = 0.032), but not with CBF (Spearman's *ρ* = −0.044, *P* = 0.863). Note that only cortical microbleeds were assessed as hypointensities on MGE sequences in deeper structures of the brain are less specific and may represent mineralisation, previously observed in aged WT mice.^26^

### Arteriolar SMA is decreased in 18- and 24-month-old APP23 Tg mice

**Figure 7:**
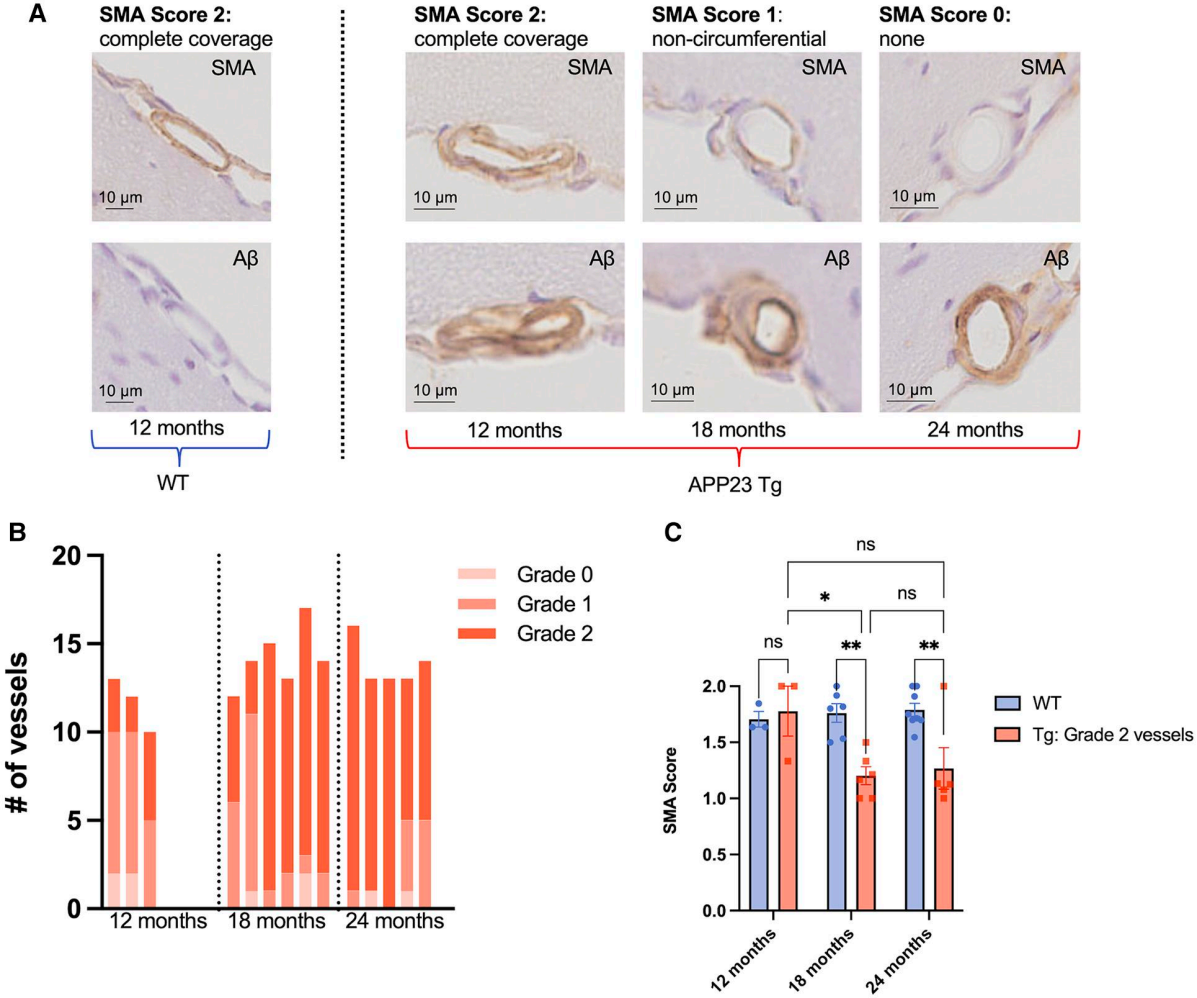
**SMA is decreased in arterioles with vascular amyloid-β in 18- and 24-month-old APP23 Tg mice.** (**A**) SMA and amyloid-β stains from a representative SMA score 2 vessel from a WT mouse and representative SMA score 2, 1 and 0 vessels from APP23 Tg mice. (**B**) Numbers of Vonsattel Grade 0, 1 and 2 pial arterioles identified in APP23 Tg mice of different ages. Each column represents a separate mouse [1 brain section analyzed per mouse; 12 months: 3 mice (3 female), 18 months: 6 mice (3 female) and 18 months: 5 mice (3 female) respectively]. (**C**) Average pial vessel SMA score per mouse [12 months: 3 WT mice (2 female), 3 Tg mice (3 female); 18 months: 7 WT mice (3 female), 6 Tg mice (3 female); and 24 months: 8 WT mice (3 female), 5 Tg mice (3 female)]. For APP23 Tg mice, only SMA scores from Vonsattel Grade 2 vessels were used for analysis. Two-way ANOVA demonstrated a significant age × genotype interaction (*P* = 0.044, *F* = 3.6) and significantly lower SMA scores in Tg mice (*P* = 1.9E−3, *F* = 12.1), pre-selected comparisons performed using Šídák correction shown (* *P* < 0.05, ** *P* < 0.01).

A subset of mice was selected for immunohistochemistry including staining of serial sections for amyloid-β and SMA. Pial arterioles were rated on both the Vonsattel grade of individual vessel CAA severity and SMA score (blinded to age and other stains, Fig. 7A). Note, few arterioles were observed to be Grade 0 (no amyloid-) in all Tg mice, and low numbers of Grade 1 (incomplete amyloid-β coverage) were observed in 18-month and 24-month-old Tg mice (Fig. 7B). Most vessels in 18-month and 24-month-old age groups were Grade 2 (circumferential amyloid-β). No amyloid-β was observed in WT animals (all ‘Grade 0’ vessels). SMA scores for all arterioles of each grade assessed were averaged within each mouse to generate vessel grade specific SMA scores (Fig. 7C).

Given the low numbers of Grade 0 and Grade 1 vessels in Tg mice across the age groups, only Grade 2 vessels from Tg mice were included in analysis of SMA coverage. SMA scores from Grade 2 vessels are shown in Fig. 7C. Note, all vessels assessed in WT mice were Grade 0 (no amyloid-β). A two-way ANOVA demonstrated a significant age × genotype interaction (*P* = 0.044, *F* = 3.6) and significantly lower SMA scores in Tg mice (*P* = 1.9E−3, *F* = 12.1). A post-hoc analysis were performed on pre-selected comparisons using a Šídák correction. SMA scores in Grade 2 pial arterioles was observed to be significantly lower in 18-month-old Tg mice as compared to 18-month-old WT mice (*P* = 4.5E−3) and in 24-month-old Tg mice as compared to 24-month-old WT mice (*P* = 7.5E−3). 18-month-old Tg mice had significantly lower SMA scores than 12-month-old Tg mice (*P* = 0.021). There was no significant difference between SMA coverage in 12-month-old WT arterioles versus 12-month-old Tg Grade 2 arterioles.

## Discussion

In this work, we assessed several measures of vascular structure and function in the APP23 mouse model of amyloidosis, using both *in vivo* 2-photon microscopy and MRI. An early loss of spontaneous vasomotion at the level of individual arterioles was detected with 2-photon microscopy, occurring on a similar timeline as the appearance of vascular amyloid-β (at ∼8–10 months of age). Baseline arteriolar diameter and pulsatility typically increased after these initial vascular changes (Supplementary Fig. 5). Given VSMC are essential to both vasomotion^8,9^ and maintaining cerebrovascular tone,^27^ these results suggest VSMC dysfunction begins to occur surrounding the time of initial amyloid-β deposition.

Prior studies have suggested that spontaneous vasomotion and functional hyperaemia, both dependent on VSMC function, are important for glymphatic clearance (including amyloid-β) from the brain.^9,10^ Given initial amyloid-β deposition is thought to occur ∼30 years prior to the development of symptoms in patients with CAA,^28^ the identification of vasomotion as an early biomarker for VSMC dysfunction, preceding VSMC loss, may provide a critical treatment window for interventions targeted at enhancing vasomotion or neurovascular coupling to drive glymphatic clearance. We observed that despite evidence of VSMC dysfunction, CVR (as measured by the response to inhaled CO_2_) was not yet altered in 12-month-old Tg mice. Additionally, we observed that VSMCs expressing SMA (a contractile protein) were still present in pial arterioles with circumferential amyloid-β in 12-month-old Tg mice. Similarly, a prior study of APP/PS1 mice demonstrated that while global amyloid-β burden was associated with VSMC loss, local amyloid-β deposition was not tightly linked to VSMC loss at an individual vessel level,^9^ suggesting a potential role for other factors such as soluble amyloid-β in VSMC dysfunction/loss. Further supporting this observation, in the vasomotion LME model with the best fit, we found that the % local CAA coverage did not have a significant effect on vasomotion (but did have an effect in models not including an age × genotype interaction). This finding suggests again that there may be other, potentially more global, factors driving the observed local decline in vasomotion. However, we note that a prior study of Tg2576 mice (another model of amyloidosis) at 8 months of age did not demonstrate any impairments in VSMC function despite the presence of increased soluble amyloid-β, and VSMC function was subsequently impaired in older mice with vascular amyloid-β deposition.^29^

Importantly, our finding of an intact hypercapnic CBF response in 12-month-old Tg mice suggests that some segments of the microvasculature maintain the ability to vasodilate despite initial amyloid-β deposition. Therefore, interventions aiming to enhance/drive vascular reactivity may be effective despite the loss of spontaneous vasomotion. However, a limitation to this study is that we were unable to determine which individual vessels were dilating in response to a CO_2_ challenge in the MRI portion of the study. Therefore, it remains a possibility that these findings reflect intact vasodilation at the capillary/diving arteriolar level, while the loss of vasomotion we observed with 2-photon microscopy was at the pial arteriolar level. Of note, a prior study from our group of 8–10-month-old APP/PS1 mice (another model of amyloidosis) demonstrated intact vasomotion with impaired vascular reactivity in response to visual stimuli.^9^ Future studies are needed to determine whether and when sensory stimulus-evoked vascular reactivity is affected in APP23 mice; it is possible that pathways dependent on neurovascular coupling are more profoundly affected by vascular (and parenchymal) amyloid-β accumulation.

We also observed increases in beat-to-beat pulsatility of the arterioles with age in both WT and APP23 Tg mice, increases which were potentially more pronounced in APP23 Tg mice. A more pronounced increase in pulsatility with age has been previously observed in APP/PS1 mice.^30^ Increases in beat-to-beat pulsatility in small vessels with age have also been observed in human subjects,^31^ irrespective of the presence of small vessel disease.^15^ These increases in pulsatility with age may reflect stiffening of more upstream vessels (e.g. aorta),^32^ possible increased compliance in the cerebral vasculature (another marker of VSMC dysfunction), and/or decreases in brain tissue stiffness.^33^ While prior findings have suggested a link between arteriolar pulsations and glymphatic fluid transport,^14^ a study in a mouse model of hypertension demonstrated that alterations/increases in vessel wall pulsatility may actually disrupt perivascular clearance.^34^

Reduced vasomotion occurred before CBF and CVR reductions were detectable with MRI. Notably, a CBF reduction has been previously reported in APP23 Tg mice at a similar age.^35^ The reduced CVR in response to the strong CO_2_ stimulus in 24-month-old APP23 Tg mice is particularly noteworthy, given the lower baseline CBF in this group. As CVR is measured relative to baseline CBF, one would expect confounds due to a lower baseline CBF to result in an enhanced relative CVR, rather than a diminished CVR as was observed. A significant correlation was found between microbleed count and CVR; however, it is unclear whether these are causally related or just independent results of CAA pathology.

This study also suggests that haemorrhage occurs at a later stage of disease progression than reductions in vasomotion in APP23 Tg mice. Microbleeds were observed in 2/5 mice at 12 months, 4/7 mice at 18 months, and 5/6 mice at 24 months. This timeline of microbleed development corresponds well with a prior MRI study of APP23 mice which first observed microbleeds at 16 months of age.^17^

The observed increases in baseline arteriolar diameter with age in APP23 mice as measured with 2-photon microscopy and decreases in CBF as measured with MRI are counter-intuitive. Vessel diameter increases with no change in RBC velocity would typically be expected to lead to increased CBF. Notably, local vasodilation caused by fibrillar amyloid-β on pial arteries, as observed here, has been previously reported.^36^ However, some studies have shown that amyloid-β causes vasoconstriction,^37,38^ including a recent study of dissected and pressurized pial arteries from 18-month-old APP23 mice.^39^ The different findings could be explained by the different forms of amyloid-β studied, i.e. soluble or fibrillar amyloid-β, by different vessel types affected, i.e. pial or penetrating arteries or capillaries, and/or by differences in experimental preparation. It remains possible that *in vivo* in APP23 mice, at the level of the parenchymal circulation, soluble and/or fibrillar amyloid-β may act as a vasoconstrictor. Possibly, this could lead to a loss of myogenic tone in the pial vasculature, and therefore an increase in pial vessel diameter as observed in the 2-photon data. Given the dominance of the parenchymal resistance, this would lead to the CBF reduction observed with MRI. Alternative/additive explanations for lower observed CBF in APP23 mice include decreased vasodilation of arterioles in APP23 mice in response to isoflurane anaesthesia, which was used for the MRI experiments but not for the 2-photon experiments. Decreased vasodilation to isoflurane anaesthesia has also been observed in Tg2576 mice at advanced ages (19 months), which led to relatively increased CBF in WT as compared to aged Tg2576 mice.^29^ Other considerations may be reduced capillary density (previously reported in APP23 mice^40^) and/or possible reduced neural activity at advanced ages in APP23 mice due to the high burden of parenchymal amyloid-β plaques. In general, baseline CBF is not considered a sensitive marker for CAA. CBF has been observed to be unchanged in some studies^6^ or mildly reduced in others.^7,41^ Interestingly however, in the healthy elderly population, the presence of cortical microbleeds has been associated with lower CBF.^42^

An unusual finding in this study is the increase in baseline CBF in WT mice with age. This may be related to a batch effect: all 12-month-old animals were bred and aged at Jackson laboratories, and the other age groups were a mix of in-house-bred and Jackson-bred. Although speculative, it is possible that stress during transportation led to endothelial dysfunction, which has been previously reported.^43,44^ This could lead to reduced vasodilation upon isoflurane administration, and thus lower CBF in the Jackson-bred mice. Importantly, between genotypes, the ratio of Jackson-bred and in-house-bred mice was the same, indicating that the CBF and CVR reductions in Tg mice are not biased by batch effects.

There are several limitations in this study. The group sizes in the different cohorts were relatively small and sex was not always equally distributed among the groups. Furthermore, the small group sizes made it impossible to draw conclusions on the influence of sex on the outcomes of the study. However, no obvious separation was apparent between females and males in the assessed measures of vascular function, although prior studies have noted a higher amyloid-β plaque load (both vascular and parenchymal) in female APP23 mice.^45^ Of note, the APP23 mouse in which we observed no amyloid-β plaques throughout the study was male. Another limitation is the cranial window surgery in the 2-photon cohort which may impact vascular function. Notably, mice were allowed to recover for at least 4-weeks post-surgery prior to imaging sessions, with a goal of minimising the acute effects of the surgery on neurovascular function. Additionally, we did not control for potential confounders such as behaviour, arousal state, locomotion and other types of movement, which have been shown to induce neurovascular coupling and therefore likely impacted our vasomotion measurements.^46-48^ However, we note that APP23 mice have previously been observed to be hyperactive,^49^ which would be expected to increase vasomotion (not decrease as was observed in this study). Lastly, the use of isoflurane in the MRI cohort may have influenced the results. However, the fact that a combination of imaging modalities was used in this study, each with a different set of limitations, strengthens the findings, and underscores that vascular dysfunction is a prominent early feature of the APP23 mouse model.

To conclude, this study found an early loss of vasomotion in APP23 mice, occurring at approximately the same time as vascular amyloid-β deposition, before the loss of VSMCs, reduction of CVR, and occurrence of cortical microbleeds. Detection of these changes in vasomotion may present an opportunity for early, pre-symptomatic diagnosis of CAA. Additionally, given the importance of vasomotion for glymphatic amyloid-β clearance, preserving vasomotion in the pre-symptomatic phase of the disease may be an important target for prevention studies in CAA. Future studies should be considered to determine whether this early loss of vasomotion precedes initial amyloid-β deposition.

## Supplementary Material

fcaf186_Supplementary_Data

## Data Availability

All raw data supporting the findings of this study will be made available upon reasonable request to the corresponding author (S.J.v.V.). Processed data available here: https://dataverse.harvard.edu/dataverse/Kozberg. MATLAB and R code used for analysis provided here: https://github.com/Mariel-Kozberg/APP23/.
